# Outcomes of Pediatric Orthopedic Management of Ambulatory Cerebral Palsy Utilizing a Closely Monitored, Lifespan-Guided Approach

**DOI:** 10.3390/children12091252

**Published:** 2025-09-17

**Authors:** Zhe Yuan, Nancy Lennon, Chris Church, Michael Wade Shrader, Freeman Miller

**Affiliations:** Department of Orthopedics, Nemours Children’s Health, Wilmington, DE 19803, USA; jerry.yuan@nemours.org (Z.Y.); nancy.lennon@nemours.org (N.L.); chris.church@nemours.org (C.C.); wade.shrader@nemours.org (M.W.S.)

**Keywords:** cerebral palsy, adulthood, motor function, walking activity, quality of life, pain, social participation

## Abstract

**Background:** Cerebral palsy (CP) is a static, non-progressive brain pathology that affects mobility and musculoskeletal health. **Objective:** This review aims to describe the pediatric orthopedic management strategy at one specialty center with focus on optimal lifelong mobility function for ambulatory CP. **Methods:** Beginning in the 1990s, a protocol was developed to proactively monitor children with surgical or conservative interventions. After three decades, we undertook a prospective institutional review, board-approved 25–45-year-old adults callback study. Inclusion criteria were all children treated through childhood who could be located and were willing to return for a full evaluation. **Results:** Pediatric orthopedic interventions focused on regular surveillance with proactive treatment of progressive deformities. When function was impacted, we utilized multi-level orthopedic surgery guided by instrumented gait analysis. Childhood outcomes of this approach were evaluated through retrospective studies. Results show high correction rates were achieved for planovalgus foot deformity, knee flexion contracture, torsional malalignments, and stiff-knee gait. Our prospective adult callback study evaluated 136 adults with CP, gross motor function classification system levels I (21%), II (51%), III (22%), and IV (7%), with average ages of 16 ± 3 years (adolescent visit) compared with 29 ± 3 years (adult visit). Adults in the study had an average of 2.5 multi-level orthopedic surgery events and 10.4 surgical procedures. Compared with adults without disability, daily walking ability was lower in adults with CP. Adults with CP had limitations in physical function but no increased depression. A higher frequency of chronic pain compared with normal adults was present, but pain interference in daily life was not different. Adults demonstrated similar levels of education but higher rates of unemployment, caregiver needs, and utilization of Social Security disability insurance. **Conclusions:** The experience from our center suggests that consistent, proactive musculoskeletal management at regular intervals during childhood and adolescence may help maintain in gait and mobility function from adolescence to young adulthood in individuals with CP.

## 1. Introduction

Cerebral palsy (CP) is a static, non-progressive brain injury that typically occurs in childhood, affecting mobility and musculoskeletal health, with an incidence of approximately 2–3 per 1000 live births [1]. Although most children with CP survive into adulthood and have similar lifespans as people without disabilities [2,3], musculoskeletal lower extremity impairments commonly develop during phases of rapid growth, which negatively impact functional mobility. Musculoskeletal impairments are addressed with orthopedic multi-level surgery (MLS) guided by instrumented gait analysis (IGA) [4,5]. Common deformities include equinus, equinovarus, planovalgus foot posture, flexed knee gait, stiff knee gait, tibial torsion, femoral torsion, hip adduction/scissoring gait, and hip flexed gait [4]. Left untreated, these impairments can develop into severe contractures, lever-arm dysfunction, and joint deformities, often associated with muscle and joint pain, increased risk of falls, mobility declines, and a sedentary lifestyle [2,6,7,8]. While orthopedic surgical intervention is well supported, the specific approaches to treat complex multi-level deformity and the timing of such intervention is not well established [9,10].

The gross motor function classification system (GMFCS) is used to classify mobility function in people with CP [11]. It is useful for educating families, guiding treatment, providing expected trajectory of gross motor function, and is associated with a prediction of risk for musculoskeletal impairments [12]. Though the brain injury in people with CP is non-progressive, mobility and functional levels from childhood to adulthood are widely believed to decline with age [13,14,15]. Day et al. reviewed the ambulatory ability in children and adults with CP and found that people with more impaired mobility function during childhood were more likely to experience a decline compared with people with less impaired childhood mobility function [13]. Lower levels of walking ability were significantly related to diplegia (vs hemiplegia), pain, fatigue, and decreased balance [15]. In addition to functional losses, aging in people with CP is associated with muscle loss characterized by decreases in muscle volume and increases in non-contractile tissue [16].

Declines in functional mobility with age are connected to broader quality-of-life issues. Between 63% and 82% of adults with CP report back, hip, and lower extremity pain [17,18], which contribute to fatigue, decreased walking ability, and reduced social participation, all thought to worsen with age. Educational attainment among adults with CP varies widely; college completion rates range from 21% to 53% [19,20,21], while employment rates vary from 36% to 79% [19,21], both much lower compared with adults without disability.

For ambulatory adults with CP, our clinical experience suggests that early monitoring and proactive management during childhood can prevent the development of severe orthopedic deformity and allow stable gait and functional mobility with less pain interference in adulthood. This paper aims to summarize the pediatric orthopedic treatment approach and adult outcomes of a single specialty center with a proactive strategy of early and ongoing individualized treatment of lower extremity deformities in ambulatory children with CP (GMFCS I-IV).

## 2. Materials and Methods

### 2.1. Protocol Components for Proactive Management Throughout Childhood

Our clinical philosophy of close proactive monitoring for ambulatory children with CP was designed in the late 1980s and implemented in our CP specialty center from 1990 onward [22]. This protocol focuses on CP clinic visits with a pediatric orthopedic surgeon at defined intervals: every 6 months for patients aged 2 years through the end of adolescent growth and yearly thereafter until age 20 years. This framework differs from other centers where pediatric rehabilitation medicine, developmental pediatrics, or neurology may be the primary contact point. We utilize an orthopedic-centered approach with the expectation that developing contractures are identified early so that early treatment is initiated when the deformity impedes functional development. Careful attention is paid to monitoring joint range of motion, limb alignment, gross motor function, fine motor function, mobility function, and activities of daily function. A primary goal is to surgically intervene at the time of optimal benefit and minimal recurrence risk for each individual child, aiming for two MLS intervention events between the ages of 2 and 20 years. Management during the early childhood years is typically focused on conservative interventions, including physical therapy, orthoses, spasticity management, and supportive mobility devices. These conservative interventions happen alongside close monitoring during CP (orthopedic) clinic visits to identify progressive, functionally limiting fixed deformities with use of standardized range of motion, gross motor, and patient-reported outcome (PRO) assessments. Continuous improvement in gross motor skills should be expected for children functioning at GMFCS levels I and II from age 2 to 10 years [23].

MLS, guided by gait analysis, is the standard of care for children with CP requiring orthopedic surgery [4,24]. Surgical intervention is highly individualized to each child based upon serial evaluations and IGA, with intervention timed to periods of functional plateauing in childhood or to address fixed, progressive deformities that become functionally limiting, resulting in pain and/or intolerance of orthoses. Early surgery typically focuses on primarily soft-tissue corrections, usually performed between the ages of 5 and 10 years (see Supplementary Materials, Table S1). In contrast, surgery in later childhood and adolescence, at 10 years and older, is typically focused on bony corrective surgeries (see Supplementary Materials, Table S2). Several aspects of the clinical management protocol described have been evaluated with retrospective studies using childhood data from our center. The outcomes at various stages of childhood are summarized in Table S3 (see Supplementary Materials).

### 2.2. Adult Call Back Study

To evaluate outcomes of our proactive intervention strategy, an institutional review, board-approved prospective study was conducted of children treated by this protocol who were followed up as adults. From our database, we identified 645 patients treated through childhood who are now 25 years of age or older. We were able to make contact with 171 of these adults, of whom 136 (21%) were able to return for a follow-up adult evaluation The adult evaluation included IGA, [25,26] PRO [5,25] and community-based activity monitors to assess walking activity (WA) [27] in adults with CP. The inclusion criteria were ambulatory adults with CP classified as GMFCS level I–IV, aged 25 to 45 years, who were managed from childhood and adolescence at our institution, including IGA testing during adolescence [5,27]. The adolescent visit was assumed to be a final postoperative evaluation; however, this was not always the case, and in some instances, additional surgery after the adolescent visit was recommended.

#### 2.2.1. Instrumented Gait Analysis Outcomes

The instrumented gait analysis included kinematic analysis of walking collected with a 12-camera motion capture system (Qualisys, Goteborg, Sweden). Based on the Cleveland Marker set convention, 34 reflective markers were placed at anatomical positions. Each participant walked at a self-selected pace until a minimum of three representative gait cycles for each limb were recorded. The outcome variables included kinematic range of motion (hip, knee, and ankle), gait deviation index (GDI), stride length, gait velocity, and gross motor function measure (GMFM).

#### 2.2.2. Patient-Reported Outcomes

Participants were asked to complete (with or without caregiver assistance) an online demographic survey and four domains of the patient-reported outcomes measurement information system (PROMIS): (1) physical function v2.0, (2) ability to participate in social roles and activities (participation) v2.0, (3) pain interference v1.1, and (4) depression v1.0 [5,25]. The PROMIS is a rigorously constructed, generalizable, and clinically relevant set of PROs developed at the National Institutes of Health [28].

#### 2.2.3. Walking Activity

Each participant wore a research-grade activity monitor for 8 days to monitor community WA [29]. The Modus StepWatch (Edmonds, WA, USA) is a Food and Drug Administration-approved Class II medical device worn around the ankle that tracks steps by reporting total strides and temporal frequency of strides. The StepWatch was calibrated for accuracy to each individual’s gait pattern according to manufacturer recommendations [30]. The average stride data were compared with a published sample of 193 non-disabled adults from the United States, who ranged in age from 30 to 39 years [31].

#### 2.2.4. Residual Deformity

The goal of pediatric orthopedic management with our proactive protocol is to correct all deformities by the end of childhood growth; however, this does not always occur (for a variety of reasons including social determinants of health issues that delay surgery, unexpected recurrence, and surgical over/under correction). In addition, new deformities may develop during late adolescence and early adulthood. The senior surgeon evaluated residual deformity in this adult cohort using specific criteria based on gait analysis, including kinematics and foot pressure testing [26]. For objective measures, normal was considered as within ±1 standard deviation (SD), mild was defined as 1 to 3 SD, and severe was defined as greater than 3 SD from the normal mean. Residual deformities including excess hip internal rotation, excess stance phase knee flexion, stiff knee (reduced swing phase knee flexion), excess external or internal tibial torsion, equinus, planovalgus foot posture, varus foot posture, and hallux valgus were categorized as mild (not impacting function) or severe (impacting function) [26].

## 3. Results

### 3.1. Childhood Outcomes

Examination of childhood outcomes of this approach was achieved through retrospective studies over several years. Results show high correction rates (65–92%) were achieved for planovalgus foot deformity [32,33], knee flexion contracture (mild [34,35] or severe [36,37]), stiff-knee gait [38,39], in-toeing gait [40,41], out-toeing gait [42], hip adduction [22], and flexed hip [43]. Despite short-term correction of equinus [44] and equinovarus [45], recurrence rates for these deformities were relatively high (43.8%) [44], yet good long-term outcomes were achieved after repeat surgery [26]. The pediatric outcomes of our center’s management protocol are summarized in Table S3.

### 3.2. Adult Outcomes

The adult outcomes were reported from 136 adults who consented to participate, with 120 participants completing both the online questionnaire and on-site IGA [25,26]. The remaining 16 completed only the online questionnaire. The GMFCS distribution of the study group was I (21%), II (51%), III (22%), and IV (6%). The average age at the adolescent visit was 16 ± 3 years, and at the adult visit, 29 ± 3 years, with a time between visits of 13 ± 4 years [25]. The total number of orthopedic procedures was 1247 (10.4 per patient) performed during 298 (2.5 per patient) surgical events [26].

#### 3.2.1. Instrumented Gait Analysis (Table 1)

From adolescence to adulthood, the GDI (72 ± 13 vs. 72 ± 13), gait velocity (85 ± 27 vs. 78 ± 31 cm/s), and GMFM dimension D (GMFM-D) (73% ± 21% vs. 70% ± 20%) remained stable [25]. A statistically significant decline in gait velocity was observed; however, it did not exceed the minimal clinically important difference [25]. The kinematic motion of the hip, knee, and ankle during gait showed no clinically meaningful change between visits [25]. Functional mobility in adulthood was significantly predicted by adolescent function, with higher adolescent GMFM correlating with less decline in adults [25]. Compared with the minimal clinically important difference, 40% of adults showed a decrease in gait velocity, 41% in GMFM-D, and 26% in GDI, while 37% improved in gait velocity, 40% in GMFM-D, and 44% in GDI [25].

On average, there were minor changes in gait velocity, GMFM, and GDI, with significant variations. When segmenting groups into those with minimal clinically important difference (MCID) increases and decreases for the GMFM-D, GDI, and gait velocity, there were almost equal numbers of individuals with increases compared to decreases from the adolescent assessment to the adult assessment. Based on regression analysis, these changes were more complex, as demonstrated by a negative correlation between the shift in adolescent assessment and gait velocity, with a greater decrease in gait velocity observed with increasing GMFCS levels. However, the change in GDI was also negatively correlated with the adolescent’s GDI. The fact that some higher-functioning ambulatory patients experience a greater decrease in specific parameters than lower-functioning patients makes it challenging to determine definitive predictions or identify subjects who will exhibit decreased function over the defined age span [25].

**Table 1:** Comparison of gait and functional mobility between adolescent and adult visits in individuals with CP.

Variable	*n* (Limbs)	Adolescent Visit, Mean (SD)	Adult Visit, Mean (SD)	*p* Value	MCID	Normal Mean (SD)
GDI [25]	120 (239)	72 (12)	73 (13)	0.217	5	100 (10)
Stride length (cm) [25]	120	93 (23)	90 (24)	0.163	5.8%	131 (15)
Gait velocity (cm/s) [25]	120	85 (28)	79 (33)	0.033 *	9.1%	124 (18)
GMFM-D [25]	114	29 (8)	28 (9)	0.211	1.8	39
Knee flexion IC (°) [25]	120 (239)	26 (13)	28 (12)	0.081	-	4 (4)
CPPI	81 (162)	12 (40)	14 (41)	0.530	-	−9 (21)

* indicates *p* value < 0.05 indicating significant difference. CP, cerebral palsy; CPPI, coronal plane pressure index; GDI, gait deviation index; GMFM-D, gross motor function measure dimension D; IC, initial contact; MCID, minimal clinically important difference; SD, standard deviation.

#### 3.2.2. Patient-Reported Outcomes (Table 2)

According to the demographic questionnaire’s Likert scale scoring, one-third of participants reported moderate to severe joint or muscle pain, and 40% reported moderate to severe chronic pain [5]. By subgrouping into the self-reported group (SR) and proxy-reported group (PR), adults with CP showed significantly higher levels of chronic pain, 47.1% (SR) and 53.7% (PR), compared with 11.2% found in non-disabled adults, but demonstrated similar levels of pain interference in daily activities as non-disabled adults reported according to PROMIS results [5,25].

**Table 2:** Outcomes in adults with cerebral palsy compared with non-disabled adults.

	Adult Mean (SD), GMFCS I	Adult Mean (SD), GMFCS II	Adult Mean (SD), GMFCS III	MCID	Normal Mean (SD)
PROMIS Physical Function v2.0 [25]	50 (9.4)	42 (9) *	34 (9) *	2	55 (8)
PROMIS Depression v1.0 [25]	48 (8) *	49 (8) *	49 (8)	3	52 (11)
PROMIS Participation v2.0 [25]	57 (8) *	53 (8) *	49 (9)	Not available	48 (10)
PROMIS Pain v1.1 [25]	49 (10)	52 (9) *	49 (9)	4	48 (9)
Strides/Day [27]	4324 (2003)	2705 (1568) *	890 (569) *	Not available	5127 (2834)

* Indicates *p* value < 0.05 compared with published normative values. GMFCS, gross motor function classification system; MCID, minimal clinically important difference; PROMIS, patient-reported outcomes measurement information system; SD, standard deviation.

Regarding PROMIS [46], adults with CP had significantly lower physical function than non-disabled adults (41 ± 10 vs. 55 ± 8), yet higher satisfaction with social roles (53 ± 8 vs. 48 ± 10) and lower levels of depression (49 ± 8 vs. 52 ± 11) [25]. Physical function was lower in adults with CP who had more severe functional limitations (GMFCS level) [25]. At the same time, depression and pain interference were not impacted by GMFCS level [25,26]. At each GMFCS level, adults with CP were at or above the average score compared with a non-disabled reference on the Satisfaction with Life Scale (SR vs. PR vs non-disabled adults: 26.2 ± 7.0 vs. 22.5 ± 7.5 vs. 20–24) [5].

For the adults with CP who self-reported, the high school graduation rate (99%) was similar to that of non-disabled adults (92%; *p *= 0.0173), but the bachelor’s degree achievement rate (55%) was higher than non-disabled adults (37%; *p *< 0.001) [5]. Despite having more advanced education, the unemployment rate in this group was higher than the national level at 33% and was associated with a high utilization of Social Security disability insurance (33%) [5]. Within the self-reporting group, 13% required the assistance of a caregiver. For the group reported by proxy, educational levels (73% high school graduates, 0% with a bachelor’s degree) were lower than those of the general population (*p *< 0.001), and the unemployment rate was higher than the national average, at 64% [5]. Unemployment in this group was associated with high utilization of Social Security disability insurance (85%) [5]. Within the proxy-reporting group, 71% required a caregiver [5].

#### 3.2.3. Walking Activity (Table 2)

Daily WA, measured by stride count, was strongly correlated with GMFCS levels, which were more limited in adults functioning at GMFCS level III compared to those at GMFCS I/II (785 ± 591 vs. 3130 ± 1826) [27]. Significant associations were found between WA and PROMIS physical function, GDI, gait velocity, and employment [27].

#### 3.2.4. Residual Deformity

Upon adult evaluation, 35.8% had no residual deformities, 53.8% had mild deformities without pain or motor interference, and only 10.4% presented with severe deformities that impacted mobility function or caused pain [26].

## 4. Discussion

### 4.1. Early Proactive Management Prevented Progressive Musculoskeletal Deformities

This group of young adults underwent an average of 2.5 MLS events (10.4 procedures per participant) during their childhood and adolescence [25], reflecting the long-term nature of care for complex deformities in CP. The burden of care, while substantial, was distributed and aligned with periods of growth and deformity progression. Although we have not compiled an extensive list of complications incurred during surgical treatment, our review of residual deformities revealed that almost all findings showed uncorrected residual deformities, with very few surgically created deformities [26]. At long-term follow-up, high correction rates of planovalgus, knee flexion contracture (mild or severe), and stiff knee gait were obtained, consistent with previous studies [47,48,49,50,51,52]. However, the recurrence rates of initial equinus and equinovarus correction were relatively high, similar to results of other studies (4–48%) [53,54,55,56]. Higher recurrence rates for ankle equinus highlight the challenges of initial correction but were accepted due to our emphasis on avoiding ankle hyper-dorsiflexion from over-lengthening the gastrocnemius–soleus complex associated with progressive crouch. No cases of surgical over-lengthening were identified in this population. If necessary, repeated gastrocnemius recession or Z-lengthening of the Achilles tendon, with or without anterior tibialis plication, was performed, yielding good long-term outcomes [26,57]. Revision procedures for adolescents with rigid, severe foot deformities, including arthrodesis, demonstrated relatively high satisfaction (79.2–95.2%) and outcomes without pain but with limited motion, as expected [58,59,60,61]. Patients did not complain of functional deficits due to loss of hindfoot motion; however, there were also no complaints of foot pain that would suggest adjacent joint arthritis. Internal or external tibial torsion was corrected by tibial derotation osteotomy as part of MLS [62,63,64,65]. High rates of under-corrected external tibial torsion and over-corrected internal tibial torsion were observed in patients with a residual out-toeing gait. However, these had little impact on functional outcomes [26,63,66], with external tibial torsion being more acceptable [67,68,69] and compensated by mild internal hip rotation.

### 4.2. Gait and Functional Mobility Were Maintained from Adolescence to Adulthood

Close orthopedic management during childhood and adolescence improved impairments and alignment, with functional mobility and gait maintained from adolescence to young adulthood [25]. This finding aligns with Gannotti et al.’s study, which reported that 91% of adults with CP maintained or improved childhood gait abilities after MLS, achieving similar societal participation levels to those of non-disabled adults [70]. Lundh et al. also reported that young adults with CP in a surgical group had a higher gait profile score than those in a non-surgical group [71]. In contrast, other studies emphasized the deterioration of walking ability with age. A decline in walking ability was reported in 37–71% of ambulatory adults with CP, characterized by fear of falling, decline in self-care, decline in indoor walking performance, and slower walking speed [7,72,73,74]. This decline may be more prevalent in cohorts with less corrective orthopedic surgical treatment and related to more significant pain frequency and intensity, impact of pain on daily activities, physical fatigue, and reduced balance [75].

In our studies, adults with CP demonstrated functional mobility limitations and gait deviations compared with non-disabled adults, characterized by a slow speed and mildly crouched gait; however, these were maintained with no clinical progression into adulthood [25]. Stability, in this context, suggested maintenance of function rather than normalization. Variation was present with individuals who had higher functional levels in adolescence being more likely to maintain or improve functional mobility compared with those with lower functional levels in adolescence [25,76]. Similar results were reported in Hanna et al.’s study, demonstrating no functional decline in people functioning at GMFCS levels I and II. In contrast, a decline was noted in people functioning at GMFCS levels III and IV, with the GMFM-66 scores decreasing 4.7 and 7.8 points after ages 8 and 7 years, respectively [14].

### 4.3. Patient-Reported Outcomes Demonstrated Positives and Negatives Compared with Non-Disabled Adults

Chronic pain is common in adults with CP, previously reported at levels of 60–80% [17,18] but in our cohort present in only 50% [5]. We believe this was due to the proactive management of these individuals during childhood, which involved correcting impairments in the lower extremities and potentially decreasing the prevalence of osteoarthritis, a condition that affects 32.5% of ambulatory adults [77] and is the leading cause of pain for adults with CP [78]. This difference may also be related to the “call-back” nature of our study [5] rather than surveying patients from adult clinics, which would likely be comprised of more individuals seeking treatment for pain. Adults with CP exhibited less self-reported pain interference in daily activities than those without disability [25], possibly due to less participation in sports, as well as the relatively low rate of severe residual lower extremity deformities [26]. Despite lower rates of chronic pain compared with prior reports [17,18,79], the prevalence of 40–50% remains clinically significant and underscores the need for lifelong pain management strategies. This incidence also needs to be seen in the context of the finding that pain interference in activities of daily living was the same as that of non-disabled peers.

Previous studies have indicated poor quality of life in adulthood in those with CP, characterized by depression, limited social relationships, and lower rates of independent living [19,20,21,80]. Contrary to these findings, our adult cohort had levels of depression, satisfaction with social roles, and satisfaction with life at or above our typical values [5], a trend also noted by other authors [81,82]. This may be related to the well-established family support in this cohort, which was reflected in a higher need for caregiver assistance.

Our findings suggested that adults with CP had similar levels of education to non-disabled adults but a higher frequency of unemployment and Social Security utilization [5]. This indicates the ongoing societal barriers, such as obstacles for those with mobility impairments, as well as employment discrimination, and the need for advocacy for employment support for people with disabilities.

Access to specialized health care for adults with CP remains inconsistent. While Roquet et al. found that adults with CP underutilize rehabilitative services [81], Gannotti et al. reported that most had adequate access to general health care [83]. The lack of specialized orthopedic and rehabilitation care could contribute to functional deterioration, as musculoskeletal impairments persist across the lifespan. Additionally, a high prevalence of comorbidities in CP has been linked to worsening overall health outcomes in adulthood [3,4,79,84,85].

### 4.4. Walking Activity Was Limited

Significant limitations in WA lead to concerns of long-term mobility loss in middle and older adulthood. While adults classified as GMFCS I had similar levels WA to those without disability, adults at GMFCS III and IV had very significant limitations, walking less than 1000 steps per day [27]. These WA levels were much lower than published values for non-disabled adults [31].

Comparisons of WA during childhood across GMFCS levels show decreases corresponding to GMFCS level (GMFCS I: 5600 strides per day, GMFCS II: 4650, and GMFCS III: 2050), all significantly lower compared with the 7200 strides taken by typically developing peers [86]. Althoff et al. reported that WA in people with CP decreased moderately with increasing age [87]. We observed a similar trend in adults with CP, with strong correlations between stride count and physical capacity measures, including PROMIS physical function scores and gait velocity. WA was significantly associated with employment consistent with research on the adult workforce that finds individuals with physically demanding jobs engaged in higher levels of WA [88]. In our cohort, employment and WA activity declined with increasing GMFCS levels, highlighting the correlation between ambulatory ability and employment.

This current retrospective review focused only on the impact of surgical management, as we lacked the means to evaluate the effects of multiple therapies that these patients also encountered, which also impacted the outcome [89]. There is also a psychological impact of cerebral palsy that we recognize; however, we also did not have measures to evaluate the effects of interventions that were received by this population [90].

We acknowledge that our adult callback study, which was mainly based on a single center’s studies, may be subject to selection bias, as only those who were willing and able to return (136 of 171 contacted individuals) were included. An attempt was made using all our hospital records, social media, and internet searches, and we were able to find only 171 out of the 645 eligible individuals. Those who returned may represent individuals with more favorable experiences or greater family support. This may skew results toward better functional and psychosocial outcomes and limit generalizability. In addition, only eight patients were classified as GMFCS IV, which did not provide statistical significance, especially for the comparison of gait and functional mobility between adolescent and adult visits. Furthermore, without comparing the outcomes of patients who didn’t receive any surgical treatments, we could not confirm whether the changes in outcomes were due to the treatment or the natural history of these patients.

## 5. Conclusions

Early proactive musculoskeletal management throughout childhood helps to maintain gait, walking ability, long-term mobility, and quality of life in ambulatory young adults with CP. Future research is needed to examine the orthopedic status and mobility function of adults into middle age and older adulthood. Advocacy efforts should focus on enhancing social participation, supporting employment, and improving access to independent living environments for adults with CP.

## Data Availability

Data are available by request from the corresponding author due to legal reason.

## Supplementary Materials

The following supporting information can be downloaded at: https://www.mdpi.com/article/10.3390/children12091252/s1, Table S1. Surgical recommendations for patients 5–10 years old; Table S2. Surgical recommendations for patients 10 years old and older; Table S3. Childhood outcomes of specific surgical procedures at our specialty center.

## Abbreviations

The following abbreviations are used in this manuscript:

CP	Cerebral palsy
GDI	Gait Deviation Index
GMFCS	Gross Motor Function Classification System
GMFM	Gross Motor Function Measure
GMFM-D	Gross Motor Function Measure Dimension D
GMFM-66	Gross Motor Function Measure 66
IGA	Instrumented Gait Analysis
MLS	Multi-level surgery
PR	Proxy-reported
PRO	Patient-reported outcome
PROMIS	Patient-Reported Outcomes Measurement Information Systems
SD	Standard deviation
SR	Self-reported
WA	Walking activity
