# Supplementary material for: Outcomes of Pediatric Orthopedic Management of Ambulatory Cerebral Palsy Utilizing a Closely Monitored, Lifespan-Guided Approach
Source: Children (Basel). 2025 Sep 17;12(9):1252. doi: 10.3390/children12091252 (PMC12469097; doi:10.3390/children12091252)
Supplement: Supplementary file 1 [file children-12-01252-s001.zip › children-3819915-supplementary.pdf]

Supplementary Materials:

Table S1. Surgical recommendations for patients 5-10 years old.

Deformity	Physical Examination	Kinematics	Pedobarograph	GMFCS Pattern	Treatment	Precaution
Equinus toe walking	Dorsiflexion knee extended <0°, but normal with knee flexed	Zero dorsiflexion in stance	No or minimal heel contact	II & III bilateral	Gastrocnemius recession	Be very careful to avoid overlengthening, prevent lengthening the soleus
Equinus toe walking	Dorsiflexion knee extended <0°, same with knee flexed	Zero dorsiflexion in stance	No or minimal heel contact	I or II unilateral	Open Z-lengthening of the Achilles tendon	Do not allow severe fixed equinus to develop, which leads to cavus foot deformity
Equinovarus	Lack dorsiflexion	No dorsiflexion in stance	No heel contact and all lateral forefoot weight bearing	I, II, or III unilateral or bilateral	Only address the gastrocnemius or Achilles based on the severity of equinus	Avoid tibialis posterior surgery under age 8 or 9 yrs due to high risk of over-correction
Planovalgus	Passive correctable planovalgus, if dorsiflexion (< -20°)	Zero dorsiflexion	No heel contact	II or III bilateral, or WGH IV unilateral	Consider gastrocnemius lengthening, especially if not tolerating ankle-foot orthosis	Avoid early bone foot surgery due to high recurrence risk
Stance knee flexion	Unilateral popliteal angle >60°, or knee flexion contracture <10°	More than 25° knee flexion at foot contact and knee flexion above normal in mid stance		I, II, or III unilateral or bilateral	Distal hamstring lengthening--medial only if normal or internal tibial torsion, include lateral if external tibial torsion or popliteal angle severe (>80°)	Avoid overlengthening, should have a residual popliteal angle under anesthesia of 30°
In-toeing gait	extended hip external rotation (<0°) or internal tibial torsion (>20°)	Foot progression angle >45° internal	>45° internal foot progression	I & II unilateral more likely to intervene to improve gait	For the very severe in-toeing can consider femoral or tibial derotation, usually try to delay until age 9 yrs or older to prevent recurrence	Do not over-correct internal femoral torsion & do not over-correct internal tibial torsion because with growth, there will be increased external torsion

adducted hip, scissoring gait	Check hip x-ray, usually torsional malalignment	<10° hip abduction (with hip extended) and normal hip rotation		II or III bilateral, or WG IV unilateral	Open adductor longus and gracilis release	If slight over-correction, it will recover with growth, although severe over-correction may not correct with growth
Flexed hip	Severe hip flexion contracture (>40°)	Maximum stance extension (<40°)		III bilateral	iliopsoas intramuscular lengthening	Avoid complete iliopsoas release in ambulators, which leads to permanent hip flexor weakness

GMFCS, Gross Motor Function Classification System; WGH, Winters, Gage, and Hicks classification.

Table S2. Surgical recommendations for patients 10 years old and older.

Deformity	Physical Examination	Kinematics	Pedobarograph	GMFCS Pattern	Treatment	Precaution
Equinus toe walking	Dorsiflexion knee extended <0°, but normal with knee flexed	Zero dorsiflexion in stance	No or minimal heel contact	II & III bilateral	Gastrocnemius recession, if severe (< -20°) dorsiflexion - anterior tibial plication	Avoid over-lengthening, especially avoid soleus lengthening
Equinus toe walking	Dorsiflexion knee extended <0°, same with knee flexed	Zero dorsiflexion in stance	No or minimal heel contact	I or II unilateral	Open Z-lengthening of the Achilles tendon, if severe (< -20°) dorsiflexion- anterior tibial plication	Even for severe contractures with cavus, correct midfoot cavus, which may appear as equinus
Equinovarus	Lack dorsiflexion	No dorsiflexion in stance, can correct varus passively	No heel contact and all lateral forefoot weight bearing	I or II unilateral or bilateral	Gastrocnemius or Achilles based on severity of equinus, split tibialis posterior to peroneus brevis, or split tibialis anterior transfer to peroneus longus, mild varus, may do intramuscular length tibialis posterior	For severe stiff varus, calcaneal osteotomy or lateral calcaneal shortening may be required
Planovalgus feet	Planovalgus with medial arch collapse	Usually has external foot progression, may cause crouch due to lever arm dysfunction	Most weight bearing on the medial mid-foot and forefoot	II & III bilateral	GMFCS I & II mild to moderate planovalgus--lateral calcaneal lengthening osteotomy & medial column osteotomy, for GMFCS III and severe planovalgus--subtalar fusion and	Make sure the medial column is stable after correction, fusion may need to extend to medial cuneiform or first metatarsal in severe cases, especially with midfoot break, check equinus and

					medial-foot fusion as needed	tibial torsion after correction
Stance knee flexion (mild crouch)	Unilateral popliteal angle >60° or knee flexion contracture <10°	More than 25° knee flexion at foot contact and knee flexion above normal in mid-stance		I, II, or III unilateral or bilateral	Distal hamstring lengthening - Medial only if normal or internal tibial torsion, include lateral if external torsion or popliteal angle severe (>80°)	Avoid over-lengthening to prevent back kneeing in stance
Stance knee flexion (severe crouch)	Knee flexion contracture (>20°)	Knee flexion in stance (>30°)	Either no heel contact or prolonged heel contact	II or III bilateral	If 20-30° flexion contracture and significant growth remaining, use guided growth or posterior knee capsulotomy--if mature or >30° contracture, do distal femoral extension osteotomy	Make sure to shorten femur while doing the extension osteotomy to prevent sciatic nerve palsy, don't forget to do patellar tendon plication
Stiff knee gait	Spastic rectus, or rectus contracture Ely test <80°	Gait speed more than 60 cm/sec, low &/or late peak knee flexion in swing	Toe drag	II or III bilateral, some II, unilateral	Distal rectus femoris resection (remove 4-6 cm tendon) or distal rectus femoris transfer	Do not leave any rectus muscle attached to the quadriceps tendon, it will hypertrophy and again limit swing knee flexion
In-toeing gait	Extended hip external rotation less than internal or internal tibial torsion more than 10° external	Foot progression angle >0° internal	>0° internal foot progressio n	I, II, & III unilateral & bilateral improve gait and cosmesis	Femoral derotation--aim for equal internal and external extended hip rotation, tibia derotation to zero thigh-foot angle (do not rotate to external rotation because this will get worse postoperatively over time)	Do not over externally rotate, mild residual internal rotation is better than external rotation
Out-toeing gait	(>25°) External tibial torsion or external thigh-foot angle (>20°)	>30° External tibial torsion in stance phase, external foot progression	External foot progressio n and/or planovalg us	II or III bilateral	If significant planovalgus is present, correct first; if the thigh-foot angle is >20° external, add tibia derotation; if there is also internal femoral torsion, correct as the first procedure, then the feet, then the tibia	Aim for a neutral foot-to-thigh angle, err to mild internal rotation since the force of gait will tend to force into external rotation
Adducte d hip,	Check hip x-ray, usually	<10° abduction		II or III bilateral, or	Adductor lengthening is	Avoid over-correction, since

scissoring gait	torsional malalignment	(with hip extended) and normal hip rotation		WGH IV unilateral	usually not needed if the femur is derotated, but if severe or with hip dysplasia, do open adductor longus and gracilis release	there will be little self-correction after the completion of growth
Flexed hip	Severe hip flexion contracture (>40°)	Maximum stance extension (< -40°)		III bilateral	If the femurs are derotated, some proximal hip extension can be added to the osteotomy, may add iliopsoas intramuscular lengthening	Avoid releasing iliopsoas tendon, which will leave a weak hip flexor

GMFCS, Gross Motor Function Classification System; WGH, Winters, Gage, and Hicks classification.

Table S3. Childhood outcomes of specific surgical procedures at our specialty center.

**Deformity**	**GMFCS Pattern**	**Treatment**	**Good and Fair Outcomes**	**Poor outcomes**	**Complications/ related risk factors**
Equinus toe walking	II & III bilateral	Gastrocnemius recession, if severe (< -20°) dorsiflexion - anterior tibial plication	Ankle passive dorsiflexion, heel impulse, and CPPI significantly increased [44]	43.8% recurrence overall; hemiplegia (62.5%), followed by diplegia (55.7%), and quadriplegia (17.0%). No hyperdorsiflexed calcaneus posture [44]	Age at surgery has a significant influence on recurrence
Equinus toe walking	I or II unilateral	Open Z-lengthening of tendon Achilles, if severe (< -20°) dorsiflexion-anterior tibial plication			
Equinovarus	I or II unilateral or bilateral	Gastrocnemius or Achilles based on severity of equinus, after age 8 yrs, split tibialis posterior to peroneus brevis, or split tibialis anterior transfer to peroneus longus.--mild varus, may do intramuscular length tibialis posterior	32.1% good outcomes with neutral feet; 21.4% fair outcomes with mild varus and valgus feet [45]	22.9% poor outcomes with severe varus feet; 23.6% poor outcomes with severe valgus feet [45]	Diplegic, quadriplegia, younger than 8 years, and not capable of community ambulation were the risk factors for poor outcomes [45]
Planovalgus feet	II & III bilateral	GMFCS I & II mild to moderate planovalgus-lateral calcaneal lengthening osteotomy & medial column osteotomy, **FOR** GMFCS III and severe planovalgus, subtalar fusion and medial-foot fusion as needed	126/138 (91.3%) good outcomes with CPPI, medial midfoot, heel impulse improvement [32]	12/138 (8.7%) recurrence [32]	Age at the first surgery, body mass index, and increased forward velocity could predict recurrence [80]; 10/43 (23.3%) underwent hardware removal;

					9/43(20.9%) had foot pain and older, poor functional abilities, and subtalar fusion developed less pain [33]
Stance knee flexion (mild crouch)	I, II, or III unilateral or bilateral	Distal hamstring lengthening--medial only if normal or internal tibial torsion, include lateral if external torsion or popliteal angle severe (>80°)	Popliteal angle, knee flexion at foot contact, and GDI were significantly improved without back-kneeing gait at maturity [34]	Average knee flexion remained unchanged [34]; decreased hip peak power generation [35]	Older age, male, and concomitant plantarflexor lengthening predicted more knee flexion [34]
Stance knee flexion (severe crouch)	II or III bilateral	If 20-30° flexion contracture and significant growth remaining, use guided growth or posterior knee capsulotomy; if mature or >30° contracture, do distal femoral extension osteotomy	For posterior knee capsulotomy, 38/59 (64.4%) knee flexion contracture had significantly improved [36]; significant improvement in knee flexion contractures and GDI [37]	21/59 (35.6%) had recurrence [36]; gait velocity and stride length remained unchanged in the posterior knee capsulotomy group and decreased in the distal femoral extension osteotomy group [37]	Capsulotomy group 7/59 (11.9%)-6/35 (17.1%) transient sciatic nerve palsy; 2/59 (3.4%)-1/35 (2.9%) wound dehiscence; 2/59 (3.4%) needed revision; 4/10 (40%) transient sciatic nerve palsy in the extension osteotomy group [36,37]
Stiff knee gait	II or III bilateral, some II unilateral	Distal rectus femoris resection (remove 4-6 cm tendon) or distal rectus femoris transfer to the semitendonosis	Significant improvement in peak knee flexion, knee range of motion, mean time to peak knee flexion, and reduced toe drag [38]; both rectus femoris resection and transfer showed significant improvement [39]		Compared with GMFCS I/II, children with GMFCS III underwent a greater tendency to crouch after rectus femoris transfer; Less improvement with low gait velocity (<80 cm/sec) [39]
In-toeing gait	I, II, & III unilateral & bilateral improve gait and cosmesis	FDO aims for equal internal and external extended hip rotation, TDO to zero thigh-foot angle (do not rotate into	For hip internal rotation patients, hip kinematic rotation and passive range of motion midpoint	40% (by kinematic hip rotation) and 39% (by hip midpoint) underwent hip	Higher levels of spasticity and lower gait velocity predicted long-term hip internal

		external rotation because this will get worse postoperatively over time	improved by FDO [40]; for internal tibial torsion patients, 16/36(44%) were corrected by [41] external TDO	internal rotation recurrence [40]; 20/36 (56%) over-corrected with external TDO; no under-corrections [41]	rotation recurrence [40]
Out-toeing gait	II or III bilateral	If significant planovalgus is present, correct first; if the thigh-foot angle is >20° external, add tibia derotation; if there is also internal femoral torsion, correct as the first procedure, then the feet, then the tibia	For external tibial torsion patients, 16/43 (37%) were corrected by internal TDO [42]	25/43 (58%) under-corrected, 2/43 (5%) over-corrected by internal TDO [42]	Compared with patients with GMFCS level I/II, patients with GMFCS level III had a higher rate of under-correction (72.2% vs 50%) but a lower rate of over-correction (0% vs 8.3%) [42]
Adducted hip, scissoring gait	II or III bilateral, or WGH IV unilateral	Adductor lengthening is usually not needed if the femur is derotated, but if severe or with hip dysplasia, do open adductor longus and gracilis release	43/65 (67%) had good results with decreased MP and prevention of hip dislocation [22]	22/65 (33%) had poor results, and 19/65 (30%) required hip reconstruction [22]	Spastic diplegia, walkability MP at 1-year postoperatively, predicted a good long-term outcome [22]
Flexed hip	III bilateral	If the femurs are derotated, some proximal hip extension can be added to the osteotomy, may add iliopsoas intramuscular lengthening	For WGH type 4 hemiplegic patients, hip reconstruction improved hip morphology, relieved pain, and maintained a high functional level [43]		

CPPI, coronal plane pressure index; FDO, femoral derotation osteotomy; GDI, Gait Deviation Index; GMFCS, Gross Motor Function Classification System; MP, migration percentage; TDO, tibial derotation osteotomy; WGH, Winters, Gage, and Hicks classification.
